# Factors Associated with Liver Enzyme Abnormalities in HIV–HBV and/or HCV Co-infected Patients in Kinshasa, Democratic Republic of the Congo: Multicenter Cross-sectional Study

**DOI:** 10.5041/RMMJ.10474

**Published:** 2022-07-31

**Authors:** Jean-Paul Mayimona Kimpiatu, Charles N’lombi Mbendi, Antoine Wola Yaba Tshimpi, Aliocha Natuhoyila Nkodila, François Bompeka Lepira, Sebastien Nsukini Mbendi, Fiston Mbutiwi, Jean-Robert Rissassy Makulo, Hippolyte Nani-Tuma Situakibanza, Benjamin Longo-Mbenza

**Affiliations:** 1 Department of Gastroenterology, University of Kinshasa, Kinshasa, Democratic Republic of the Congo; 2 Department of Family Medicine and Primary Health Care, Protestant University in Congo, Kinshasa, Democratic Republic of the Congo; 3 Department of Nephrology, University of Kinshasa, Kinshasa, Democratic Republic of the Congo; 4 Department of Nephrology, University of Kikwit, Kikwit, Democratic Republic of the Congo; 5 Department of Infectious Diseases, University of Kinshasa, Kinshasa, Democratic Republic of the Congo; 6 Department of Cardiology, University of Kinshasa, Kinshasa, Democratic Republic of the Congo

**Keywords:** Co-infection, hepatitis, human immunodeficiency virus, liver enzyme abnormalities

## Abstract

**Background and Objective:**

Liver enzyme abnormalities (LEA) are extremely common and sometimes severe in individuals infected with human immunodeficiency virus (HIV), but data for this disorder are lacking in the developing countries. The objective of this study was to identify factors associated with LEA in HIV–hepatitis B virus (HBV)/hepatitis C virus (HCV) co-infected patients in Kinshasa, Democratic Republic of the Congo.

**Methods:**

This cross-sectional analytical study included 180 people living with HIV (PLWHIV) mono-infected or co-infected with HBV/HCV between November 10, 2013 and January 10, 2014 in Kinshasa. Sociodemographic, clinical, biological, serological, and immunological data were analyzed. Levels of serum glutamate oxaloacetate transferase (SGOT) and serum glutamate pyruvate transaminase (SGPT) were determined. Antibody levels were determined using enzyme-linked immunosorbent assay (ELISA).

**Results:**

The mean age of patients was 44.2±11.0 years; female sex was predominant (76.7%). Co-infection, mainly with HBV, but also HCV, was found in 43 (23.9%) patients. Elevated liver enzymes were found in 77 (42.8%) of the patients. No difference was found in the rate of liver enzyme abnormalities between patients with HIV mono-infection or HIV co-infection (46.7% versus 30.2%, respectively; *P*=0.08). Factors associated with LEA were age ≥50 years (adjusted odds ratio [OR] 2.7; 95% CI 1.4–5.5), duration of HIV infection >3 years (adjusted OR 2.7; 95% CI 1.4–5.5), and CD4 T cells count ≤303 cells/mm^3^ (adjusted OR 2.2; 95% CI 1.1–4.5).

**Conclusions:**

Liver enzyme abnormalities are frequent in patients co-infected with HIV–HBV/HCV as well as in HIV patients without co-infection. Diagnosis is determined based on age, immunodeficiency, and length of illness.

## INTRODUCTION

Liver enzyme abnormalities (LEA) are extremely frequent in patients infected with human immunodeficiency virus (HIV).^1^ Liver laboratory test abnormalities appear in more than two-thirds of these subjects.^1^ These disturbances may be due to HIV and hepatotropic virus co-infection such as hepatitis B virus (HBV) or hepatitis C virus (HCV), opportunistic infections, and drug toxicity.^2^

The coexistence of HIV–HBV/HCV co-infection and LEA is therefore frequent, especially since HBV, HCV, and HIV are risk factors for hepatic abnormalities such as hepatic cytolysis. It is recognized that any situation of immune deficiency can promote the multiplication of HBV, HCV, and HIV with deleterious consequences on the liver.^3^–^5^

In the Democratic Republic of the Congo, studies assessing LEA in people living with HIV (PLWHIV) are lacking. The objective of this study was to assess factors associated with liver enzyme abnormalities in PLWHIV co-infected with HBV/HCV in Kinshasa.

## METHODS

### Study Setting, Design, and Time Period

This was a cross-sectional and analytical study, carried out in the city of Kinshasa, the political capital of the Democratic Republic of the Congo. In this city, HIV care is organized in all general hospitals, in certain general and university hospitals, in certain private medical centers, and in the medical training of certain non-governmental organizations (NGOs). The Pediatric Foundation of Kimbondo and the Community Actions AIDS/Better Future for Orphans in Congo (AMO-Congo) are among the oldest centers in Kinshasa for HIV care. The study took place between November 10, 2013 and January 10, 2014.

Patients who are treated in these two centers are routinely administered a questionnaire that collects sociodemographic information, medical history, and at-risk behaviors for HIV. Clinical, immunological, and therapeutic information relating to HIV is collected. All patients also undergo testing for liver enzyme abnormalities and HBV or HCV co-infection.

### Study Population

The study protocol was submitted to and approved by the ethics committee of the School of Public Health of Kinshasa/DRC (ESP/CE/012/14). The present study included patients aged at least 18 years and known to be HIV carriers who consulted during the study period in one of the selected sites. Written informed consent was obtained from all participants before enrollment.

### Data Collection and Procedure

For the purposes of this study, we retrieved variables of interest including sociodemographic parameters such as age, sex, employment status, marital status, and level of education. Clinical data collected included the duration of HIV infection (years), the clinical stage of the HIV infection, the current antiretroviral therapy (ART) regimen, and recent CD4 T cells count; only CD4 T cell counts taken 3 months or less before the survey were taken into account. Blood tests were acquired for the HBV biological markers: hepatitis B surface antigen (HBsAg); hepatitis B surface antibody (anti-HBs); hepatitis B core antibody (anti-HBc); hepatitis B e-antigen (HBeAg); and hepatitis B e-antibody (anti-HBe). For HCV, total hepatitis C antibody (anti-HCV) RNA was tested. Since serum glutamate oxaloacetate transferase (SGOT) and serum glutamate pyruvate transaminase (SGPT) are markers of LEA, tests were performed to determine SGOT and SGPT levels.

### Definitions

The duration of HIV infection was defined as the time between discovery of HIV and the study. Positive anti-HBs RNA was defined as a titer of >12 IU/L^2^; co-infection with HIV–HBV was determined if HBsAg was positive, and co-infection with HIV–HCV was determined based on a positive anti-HCV test.^6^ Values for SGOT and SGPT >40 IU/L were considered abnormal.^7^

### Data Analyses

Data processing was performed using SPSS version 21 and STATA version 10.1 software. Descriptive statistics were applied to describe the rate of different demographic and clinical variables of interest (sex, marital status, employment, education, HIV clinical stage, ARV treatment, and HBV and HCV markers). Means and standard deviations were calculated for continuous variables with normal distribution. Medians and interquartile range (IQR) were calculated for non-normally distributed continuous variables. Rates for LEA in HIV patients with and without co-infection were analyzed with chi-square test for independence. To evaluate possible factors associated with LEA, patients were stratified according to different age groups (<40 years, 40–49 years, ≥50 years), duration of HIV (≤3 years, >3 years), HIV clinical stage (I to IV), and CD4 T cell counts (>303 cells/mm^3^, ≤303 cells/mm^3^). Duration of HIV and CD4 T cell counts were dichotomized at their median value.

Association of LEA with sex, duration of HIV infection, clinical stage of HIV, CD4 T cell counts, ARV treatment, and status of HIV co-infection was further evaluated with logistic regression using the step-by-step ascending Wald test. Adjusted odds ratio (OR) and the 95% confidence intervals (95% CI) were then calculated. Statistical significance was defined as *P*<0.005.

## RESULTS

**Figure 1:**
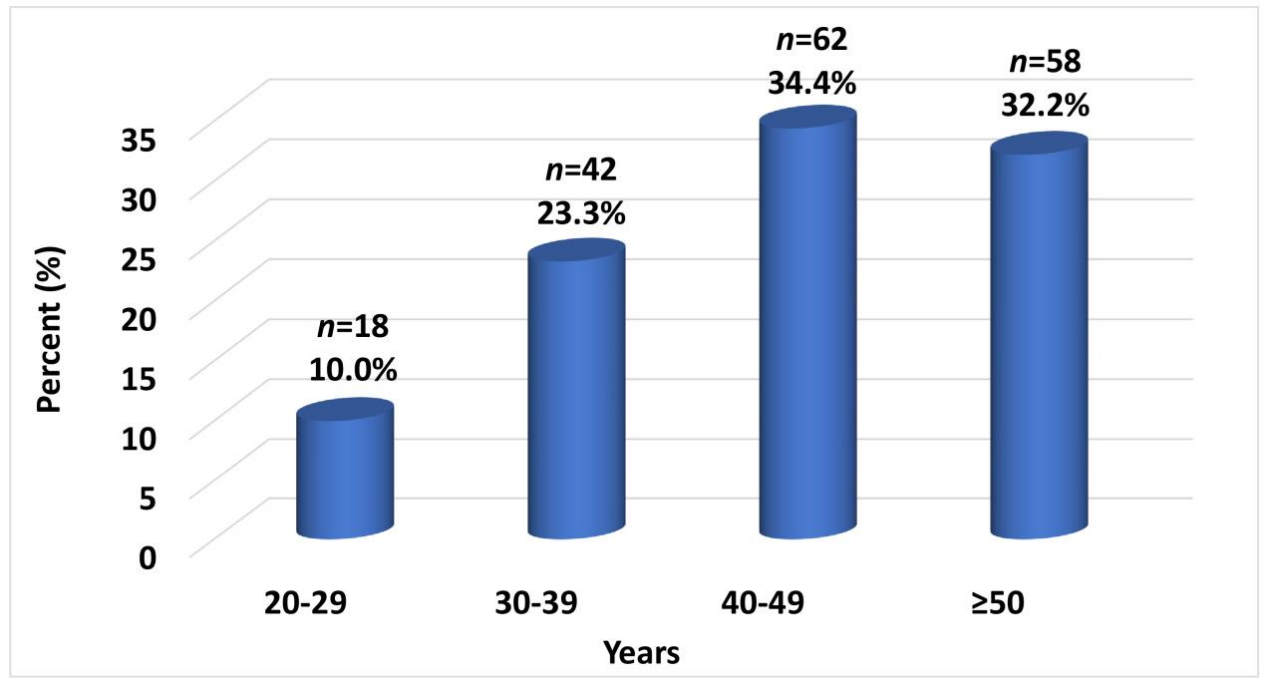
Age Distribution of Infected Study Participants.

A total of 180 HIV patients were included in the study. Their sociodemographic characteristics are reported in Table 1. The mean age of the patients was 44.2±11.0 years (Figure 1). Demographic data of particular interest were related to sex, employment status, and duration of HIV infection (median 3 years). Females outnumbered males (Table 1). Above half were unemployed. The median time of known HIV status was 3 years (range 1 to 20 years). Over half of the patients were in stage III or IV infection. The median CD4 T cell lymphocyte count was 303 cells/mm^3^ (range 2 to 1133 cells/mm^3^); 40.4% of patients had fewer than 200 CD4 T cell lymphocytes/mm^3^. At the time of the study, only 11.1% of patients were not being treated. Combination treatment with zidovudine (AZT), lamivudine (3TC), and nevirapine (NVP) was the most widely used treatment regimen.

**Table 1:** General Patient Characteristics.

Variable	Number (*n*=180)	Percent
Gender		
Female	138	76.7
Male	42	23.3

Marital status		
Married	69	38.3
Widow(er)	57	31.7
Single	32	17.8
Divorced	22	12.2

Employment status		
Unemployed	103	57.2
Self-employed	41	22.8
Official	14	7.8
Other	22	12.2

Level of education		
Primary or less	28	15.7
Secondary	129	71.5
Higher or university	23	12.8

Duration of HIV infection (years), median (IQR)	3 (1–20)	

Clinical stage of HIV infection		
I	18	10.0
II	55	30.6
III	83	46.1
IV	24	13.3

CD4 count (cells/mm^3^), median (IQR)	303 (2–1133)	

Antiretroviral therapy treatment		
No treatment	20	11.1
AZT+3TC+NVP	133	73.9
TDF+3TC+EFV	20	11.1
ABC+DDI+[Aluvia or LPV/r]	7	3.9

3TC, lamivudine; ABC, abacavir; AZT, zidovudine; DDI, didanosine; EFV, efavirenz; IQR, interquartile range; LPV, lopinavir; NVP, nevirapine; TDF, tenofovir.

**Figure 2:**
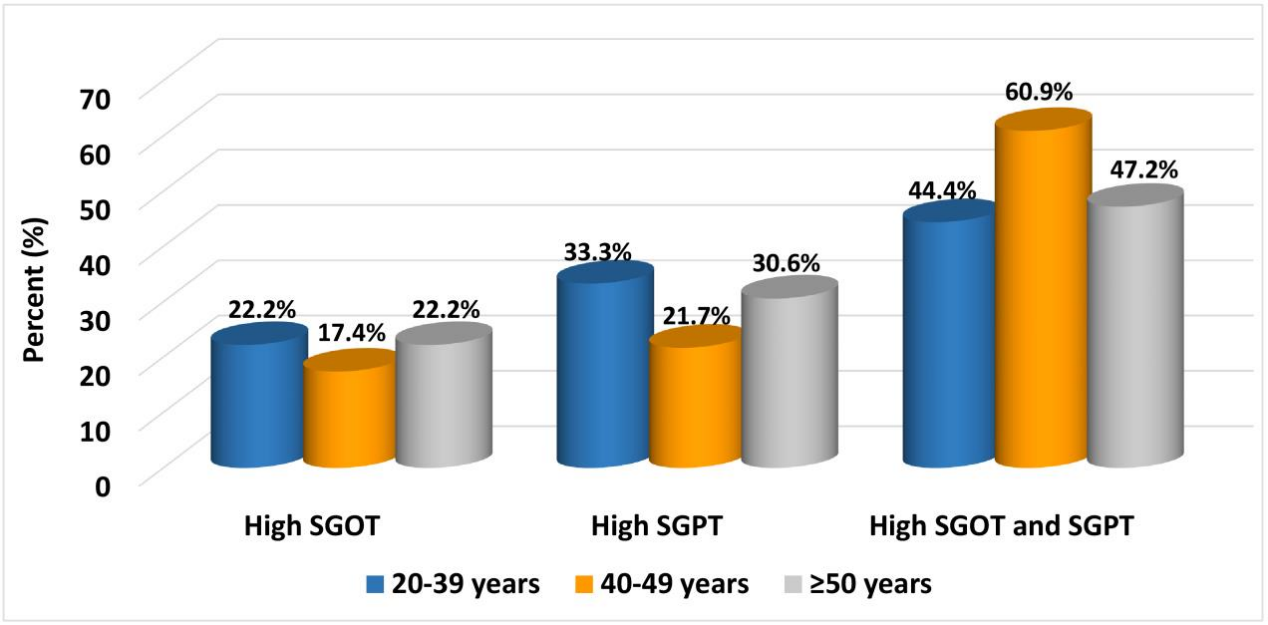
Levels of Liver Enzymes and Age Distribution.

Table 2 summarizes the serological profile of the HBV and HCV markers in the study population. Liver function abnormalities in patients according to co-infection status are presented in Table 3. Overall, 43 (23.9%) patients were found to be co-infected with hepatitis: 38 with HBV, 1 with HCV, and 2 with both HBV and HCV. Elevated liver enzymes were found in 77 (42.8%) patients. The frequency of SGOT and SGPT abnormalities increased with increasing age (Figure 2). No difference was found in the rate of liver enzyme abnormalities between patients with HIV mono-infection or HIV co-infection (46.7% versus 30.2%, respectively; *P*=0.08).

**Table 2:** Serological Profile of Patients with Hepatitis B (HBV) and Hepatitus C (HCV) Viruses.

Variable	Number (*n*=180)	Percent
**HBV Markers**		

Qualitative test (rapid)		
HBsAg positive	11	6.1

ELISA tests		
HBsAg positive	41	22.8
HBeAg positive	1	0.6
Anti-HBs+RNA	52	28.9
Anti-HBe+RNA	16	8.9
Anti-HBc+RNA	108	60.0

**HCV Markers**		

Qualitative test (rapid)		
Anti-HCV+RNA	5	2.8

ELISA test		
Anti-HCV+RNA	3	1.7

Anti-HBc, hepatitis B core antibody; anti-HBe, hepatitis B e-antibody; anti-HBs, hepatitis B surface antibody; anti-HCV, hepatitis C antibody; HBeAg, hepatitis B e-antigen; HBsAg, hepatitis B surface antigen.

**Table 3:** Liver Function Abnormalities in HIV Patients with or without HBV and/or HCV Co-infection.

Liver Function Test	Overall (*n*=180)	HIV (*n*=137)	HIV and Co-infection (*n*=43)	*P* Value
Normal, *n* (%)	103 (57.2)	73 (53.3)	30 (69.8)	
High SGOT, *n* (%)	16 (8.9)	13 (9.5)	3 (7.0)	0.080
High SGPT, *n* (%)	22 (12.2)	17 (12.4)	5 (11.6)	
High SGOT and SGPT, *n* (%)	39 (21.7)	34 (24.8)	5 (11.6)	

HBV, hepatitis B virus; HCV, hepatitis C virus; HIV, human immunodeficiency virus; SGOT, serum glutamate oxaloacetate transferase; SGPT, serum glutamate pyruvate transaminase.

Univariate analysis revealed a significant association between patient age, duration of HIV infection, clinical stage of infection, and CD4 T cell count with LEA. However, with logistic regression, only patient age, duration of HIV infection, and CD4 T cell count remained significantly associated with LEA. The risk of LEA increased linearly with patient age. This risk was higher in PLWHIV for more than 3 years (adjusted OR 2.7; 95% CI 1.4–5.5) and in those with a CD4 T cell count ≤303 cells/mm^3^ (adjusted OR 2.2; 95% CI 1.1–4.5) when compared to other parameters (Table 4).

**Table 4:** Factors Associated with Liver Enzyme Abnormalities (Univariate and Multivariate Analysis).

Associated Factor	Logistic Regression			

	Crude OR (95% CI)	*P*	Adjusted OR (95% CI)	*P*
Age (years)				
<40	1		1	
40–49	1.4 (0.6–3.1)	0.421	1.5 (0.6–3.5)	0.489
≥50	3.8 (1.7–8.8)	0.001	3.9 (1.7–9.2)	0.005

Duration of HIV infection				
≤3 years	1		1	
>3 years	2.4 (1.3–4.7)	0.004	2.7 (1.4–5.5)	0.005

Clinical stage of HIV				
I	1		1	
II	1.8 (0.5–8.7)	0.589	1.1 (0.3–2.7)	0.689
III	4.6 (1.3–20.4)	0.005	1.6 (0.3–2.4)	0.189
IV	1.4 (0.3–8.1)	0.317	1.2 (0.4–3.1)	0.569

CD4 cell count (cells/mm^3^)				
>303	1		1	
≤303	1.9 (1.0–3.9)	0.042	2.2 (1.1–4.5)	0.024

CI, confidence interval; HIV, human immunodeficiency virus; OR, odds ratio.

## DISCUSSION

The objective of this study was to assess factors associated with LEA in people mono-infected with HIV or co-infected with HBV/HCV in Kinshasa. Analysis of our patient samples showed that 22.8% were positive for HBsAg, although this seroprevalence is higher than that found by other authors in Africa, India, and Brazil, and by one other study from the Democratic Republic of the Congo, with seroprevalences in the range 8%–15%.^8^–^16^ The difference between theirs and our findings could be explained by our small study population and the fact that we used a more sensitive test. Only 1.7% of our patients had anti-HCV positive serology. This agrees with data from the literature which classify sub-Saharan Africa as a region with a low prevalence of HCV infection.^17^,^18^ On the other hand, Okoroiwu et al. recorded 8.7% patients with hepatitis C antibodies,^15^ and Mendes-Corrêa et al., in Brazil, found 17.7%.^8^ The prevalence of triple HIV-viral HBV and HCV infection was 0.6% (*n*=1), quite close to that of Platt et al. in Nigeria which was 1%,^10^ and Mendes-Corrêa et al. found 1.8%.^8^ On the other hand, this prevalence is extremely lower than that described in most studies which show a wide range of prevalences as high as 90% and as low as 10%. Still, DNA testing for HBV is necessary since the outcome for these patients has not been clearly defined.^6^,^19^–^22^

No statistical significance in the LEA rate was noted between HIV patients with or without HBV/ HCV co-infection. The LEA observed in our study may be explained by the fact that over 70% of our patients were on antiretroviral treatment containing a protease inhibitor, and the duration of HIV was at least 3 years. Our results were in agreement with the literature, since LEA increases with the duration of ARV treatment and in the presence of HBV and/or HCV co-infection. The LEA are higher in cases of triple therapy including a protease inhibitor and/or a non-nucleoside reverse transcriptase inhibitor, as compared to dual therapy with reverse transcriptase inhibitors.^23^–^26^

Univariate analysis revealed that the patient’s age, clinical stage of infection, duration of HIV infection, and CD4 T cell count were all associated with LEA; however, logistic regression showed that patient age, duration of HIV infection, and CD4 T cell count remained associated with LEA, while the clinical stage of infection did not. The association between LEA and advancing age has been shown in the literature.^27^ In older patients (≥50 years), there is an increase in the muscle enzyme creatinine kinase that accelerates muscle lysis. This muscle lysis is accentuated in the presence of HIV–HBV/HCV co-infection, thus leading to LEA.^27^ Extremely low CD4 T cell values are associated with the presence of opportunistic infections, which, after treatment, lead to immune reconstitution syndrome. This resumption of immunity is the basis of inflammation at the sites of opportunistic infections. In patients co-infected with HCV and HBV, the syndrome manifests as elevated levels of the SGOT and SGPT liver enzymes.^28^

## CONCLUSIONS

Since HIV infection may be associated with hepatitis B and/or hepatitis C infection, this could alter the natural course of HIV infection and lead to complications such as LEA. Still, LEA was as common in patients mono-infected with HIV as in those with HIV and HBV and/or HCV co-infection. Factors associated with LEA were advanced age, duration of HIV infection, and low CD4 T cell count.

## Abbreviations

3TC: lamivudine
anti-HBc: hepatitis B core antibody
anti-HBe: hepatitis B e-antibody
anti-HBs: hepatitis B surface antibody
anti-HCV: hepatitis C antibody
ART: antiretroviral therapy
AZT: zidovudine
CI: confidence interval
ELISA: enzyme-linked immunosorbent assay
HBeAg: hepatitis B e-antigen
HBsAg: hepatitis B surface antigen
HBV: hepatitis B virus
HCV: hepatitis C virus
HIV: human immunodeficiency virus
LEA: liver enzyme abnormalities
NGO(s): non-governmental organization(s)
NVP: nevirapine
OR: odds ratio
PLWHIV: people living with HIV
SGOT: serum glutamate oxaloacetate transferase
SGPT: serum glutamate pyruvate transaminase
USA: United States of America.
